# In-Field Automatic Identification of Pomegranates Using a Farmer Robot

**DOI:** 10.3390/s22155821

**Published:** 2022-08-04

**Authors:** Rosa Pia Devanna, Annalisa Milella, Roberto Marani, Simone Pietro Garofalo, Gaetano Alessandro Vivaldi, Simone Pascuzzi, Rocco Galati, Giulio Reina

**Affiliations:** 1 Institute of Intelligent Industrial Technologies and Systems for Advanced Manufacturing, National Research Council, Via G. Amendola 122D/O, 70126 Bari, Italy; 2 Department of Agricultural and Environmental Science (DiSAAT), University of Bari Aldo Moro, Via G. Amendola 165/A, 70126 Bari, Italy; 3 Department of Mechanics, Mathematics & Management, Polytechnic of Bari, Via Orabona 4, 70125 Bari, Italy

**Keywords:** agricultural robotics, precision farming, deep learning, fruit detection, multi-stage transfer learning

## Abstract

Ground vehicles equipped with vision-based perception systems can provide a rich source of information for precision agriculture tasks in orchards, including fruit detection and counting, phenotyping, plant growth and health monitoring. This paper presents a semi-supervised deep learning framework for automatic pomegranate detection using a farmer robot equipped with a consumer-grade camera. In contrast to standard deep-learning methods that require time-consuming and labor-intensive image labeling, the proposed system relies on a novel multi-stage transfer learning approach, whereby a pre-trained network is fine-tuned for the target task using images of fruits in controlled conditions, and then it is progressively extended to more complex scenarios towards accurate and efficient segmentation of field images. Results of experimental tests, performed in a commercial pomegranate orchard in southern Italy, are presented using the DeepLabv3+ (Resnet18) architecture, and they are compared with those that were obtained based on conventional manual image annotation. The proposed framework allows for accurate segmentation results, achieving an F1-score of 86.42% and IoU of 97.94%, while relieving the burden of manual labeling.

## 1. Introduction

Accurate and efficient in-field data gathering is a major requirement to increase crop monitoring and management efficiency, and improving the sustainability of agricultural processes. While manual survey and sampling by experts are currently adopted to get information on crop growth and health, this process is labour intensive, expensive and often destructive. Sampling is typically done on a limited number of plants, and measures can consequently be affected by sparsity as well as by human bias, resulting in inaccurate estimations.

Unmanned ground vehicles (UGVs) equipped with visual sensors have been recently proposed as a valuable technology to automate data acquisition over large farms, while simultaneously increasing accuracy on a narrow scale as well as reducing execution time and costs [1]. Related to this is the development of robust image segmentation techniques in order to extract meaningful information from collected data. For images captured at an orchard, this entails automatically labeling each pixel, or groups of pixels as representing fruits, trunks, branches or foliage. The parsed information can then be used in higher-level tasks such as fruit counting, plant phenotyping or crop health and growth monitoring, thus providing a rich source of information for the farmers. It can also enable further robotic operations such as automated harvesting, weeding, variable rate spraying, etc. [2].

Early work in the field of image segmentation for agricultural applications is based on hand-engineered visual features, mainly in color and texture spaces, that allow fruits to be discriminated from non-fruit regions. For instance, in [3], color and texture characteristics are used to identify green and red apples. Specifically, texture-based edge detection is combined with redness measurements and area thresholding, followed by circular fitting, in order to determine the positions of apples in the image plane. A similar approach was carried out by [4]. Both papers point out the difference between color and texture properties as means for extracting information. They show how color properties allow a pixel-by-pixel analysis of the image, while texture properties, on the other hand, allow region-based analysis. Combining feature analysis methods based on color, shape and size together, it is possible to increase the accuracy of recognition. Methods that involve the extraction of shape-based features have been also proposed to improve fruit detection [5]. Although these approaches achieve accurate results for the specific crop/data set they are designed for, they can not be easily adopted for different crops, nor can data be acquired under different conditions.

More recently, deep learning methods have been proposed to increase image segmentation accuracy and robustness for variable environmental conditions [2,6,7,8]. So far, the main drawback of deep learning is the need for massive data sets in order to train from scratch the large number of weights of the whole net. The quantity and quality of labeled data remain another major limitation. Data must be properly annotated for the network to learn its prediction task, and this is generally done manually using image labeling tools. Manual labeling is particularly demanding, especially in the case of using natural images where scenes may be highly cluttered and difficult to interpret even for an expert user. Pre-trained networks and transfer learning strategies can mitigate this problem, since they allow the knowledge from a similar domain to be transferred in order to perform new tasks [9]. Transfer learning is based on the hypothesis that the first layers of the nets similarly interpret the data, regardless of the specific goal. Hence, it is not necessary to intensively train these first layers again, since the results would be close to the initial conditions; meanwhile, only the final layers need to be retrained based on a smaller target task data set. In [8], transfer learning is proposed for apple, mango and almond recognition. Two main issues are addressed: which way is the best to initialize a network for the task (due to the differences between data captured and those that were used to feed the pre-trained network); how much training data is required to capture dataset variability. Data augmentation and its benefits are also outlined. In [7], transfer learning is used to train a network for image segmentation in vineyards, in order to identify different classes of interest (i.e., grape, trunk, canopy, pole).

Pseudo-labeling has been proposed to reduce the need for labeled data, while maintaining high network performance [10]. Pseudo-labeling employs a model that is trained on a manually labeled data set, in order to make predictions in an unlabeled data set. Then, it combines the manually and automatically-labeled data sets in order to train a new model. In a previous work [11], pseudo-labeling has been shown to further boost the performance of a pre-trained DeepLabv3+ architecture for detecting grapes in low-quality natural images.

This paper further extends prior research by proposing a novel general-purpose framework to segment pomegranate fruits in field images acquired by a ground robotic platform, using multi-stage transfer learning and pseudo-labeling techniques. Pomegranates typically mature over quite a dilated time frame, and this consequently calls for constant and long-term field monitoring, which would take great advantage of process automation techniques. Nevertheless, only a few studies can be found in the literature that apply sensor-based technologies to pomegranate orchards, and these systems mainly utilized user-supplied high-resolution images and standard image processing approaches [12,13,14,15]. Recently, [16] proposed a modified Faster R-CNN (FR-CNN) for fruit detection that was tested on five types of fruits including pomegranates.

To the best of our knowledge, this is the first study that demonstrates a ground robotic system and deep learning techniques for in-field data gathering and processing in pomegranate orchards. Notable examples of agriculture robots for fields operations in different cultivations can be found, for example, in [17,18,19].

Image acquisition is performed using a consumer-grade camera, namely an Intel RealSense D435 camera (Santa Clara, CA, USA) that is mounted onboard a tracked farmer robot that traverses the orchard. A deep learning segmentation framework is then applied for the separation of fruits from non-fruit regions using multi-stage transfer learning, whereby a pre-trained network is initially tuned on pomegranate images acquired under controlled conditions; this discrimination ability is progressively improved to segment field images with increasingly complex scenarios. Specifically, images of fruits arranged on a flat surface with a neutral background are first acquired both under controlled and natural lighting conditions. Images acquired under uniform lighting are automatically labeled, based on color thresholds in RGB space followed by morphological operations, and then used for transfer learning from a pre-trained architecture. The network is successively applied to produce accurate labels for the images acquired under natural lighting disturbances, for which standard filtering techniques do not prove effective, mainly due to the presence of shadows. These labels are, in turn, included to retrain the network, which is finally applied to segment field images. In order to further enhance the network’s performance, two strategies are also proposed to minimize false negatives and false positives. In the first case, the network is retrained after adding negative examples (i.e., images of non-fruit regions); in the second case, field images that include both fruit and non-fruit regions are included. The DeepLabv3+ [20] pre-trained architecture has been chosen for transfer learning, since it has been demonstrated to be effective for the semantic segmentation of natural images [21]. However, the proposed approach is independent of the type of pre-trained architecture; therefore, other architectures may be alternatively adopted without changing the overall framework.

Experiments performed in a commercial pomegranate orchard in Apulia, southern Italy, are presented. It is shown that the proposed approach allows for accurate segmentation results, with an F1-score of 86.42% and IoU of 97.94%, leading to classification performance that is comparable to those obtained using the same network trained by conventional manual image annotation, while relieving the burden of the time-consuming manual labeling process. When compared to other deep learning approaches using manual data set annotation (e.g., [2,16]), the proposed system shows comparable results.

It is worth noting that, although the proposed system in this study is specifically tested in pomegranate cultivations, it can be reasonably extended to other types of fruit crops for which pre-existing databases are available [22], since it requires only a limited number of labeled frames acquired from the field for refinement. It should be finally noted that the proposed segmentation framework has been proven to be effective for color images acquired by a consumer-grade RGB-D sensor. Therefore, 2D fruit detection could be used as a preliminary step before extracting additional fruit morphology information based on associated 3D data.

The research is presented in the paper as follows: the acquisition system and the image segmentation framework are detailed in Section 2; experimental results are discussed in Section 3; conclusions are drawn in Section 4.

## 2. Materials and Methods

Accurate and robust image segmentation to separate fruit from non-fruit regions is fundamental to successful fruit detection and yield estimation. This paper proposes a semi-supervised deep learning approach to automatically segment pomegranate fruits in natural images, beginning with fruit images that are acquired under controlled environment conditions. Field images are captured by consumer-grade hardware from a farm robotic vehicle, while processing is performed off-line at the end of the acquisitions.

In the rest of this section, the data acquisition platform and the experimental environment are initially described; then, the proposed segmentation method is detailed.

### 2.1. Testing Platform and Data Sets

**Figure 1:**
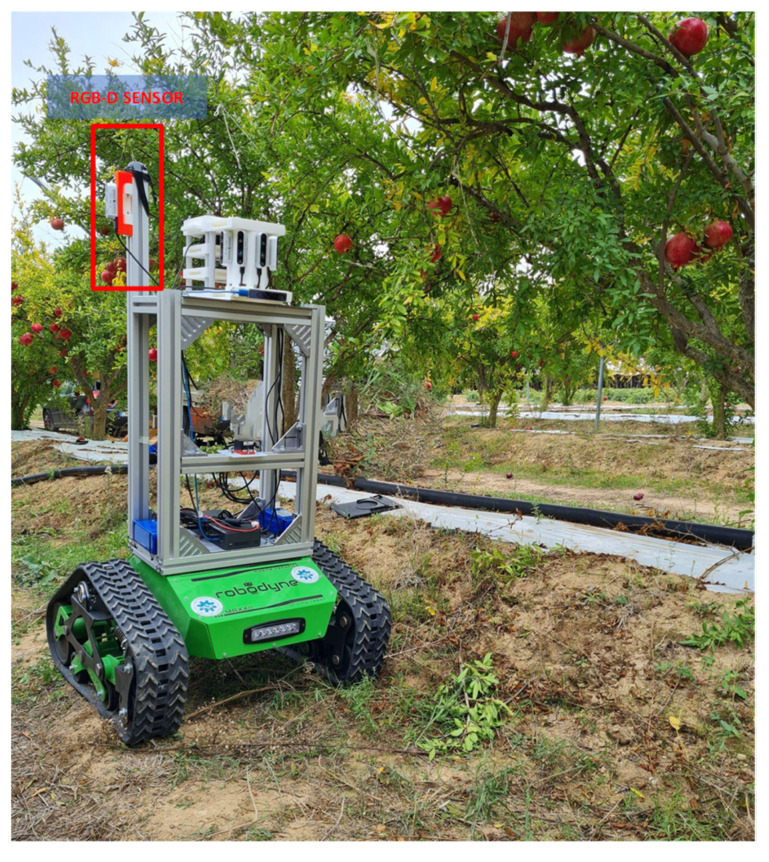
The farmer robot, Polibot, used for experimentation, equipped with a multi-sensor suite.

The testing platform *Polibot* (Figure 1) is a research ground vehicle that was completely custom-built at the Politecnico of Bari, with the aim of achieving high mobility over challenging terrain. It features an articulated suspension system that is controlled in a purely passive manner, but can fulfill high-load capacity, vibration isolation and trafficability over rough terrain, similarly to a multi-leg insect. *Polibot* has a footprint of 1.5 × 1 m and weighs about 70 kg, ensuring a payload of up to 40 kg. The control and acquisition systems have been implemented under ROS (Robot Operating System).

The *Polibot*’s standard sensor suite includes sensors to measure the electrical currents drawn by the two drive motors, in addition to an MTI 680 GNSS/INS that supports RTK cm-accuracy. The aluminum frame attached to the top plate allows the robot to be outfitted with various dedicated sensors such as laser range finders and monitoring cameras, among which an Intel RealSense D435 imaging system is used for crop visual data gathering in this study. It consists of a consumer-grade active InfraRed (IR) stereo sensor (~250 €), including a left-right IR stereo pair that provides depth information and a color camera. The color camera is a FullHD, Bayer-patterned, rolling shutter CMOS imager. Its nominal field of view is 69 (H) × 42 (V) deg, and runs at 30 Hz at FullHD. The stream acquired by the color camera is spatially-calibrated and time-synchronized with the stereo pair, so that color-aligned depth images are also available for 3D crop characterization.

Data sets were acquired at a commercial pomegranate orchard in Apulia, southern Italy, just before harvesting (October 2021). In order to collect the data sets, the vehicle was tele-operated along both sides of one row of 15 trees that were located at an average distance of about 3 m from each other. The camera was mounted about 1 m above the ground, and acquired lateral views of the tree rows from a distance of about 1.5 m. Acquisitions were performed at an image resolution of 1280 × 720 and frame rate of 6 Hz, accounting for a total of 1270 frames. Considering that the farmer robot drives at an average speed of about 0.5 m/s, a frame rate of 6 Hz was found to be a good trade-off between computational burden and pomegranate shrub coverage.

**Figure 2:**
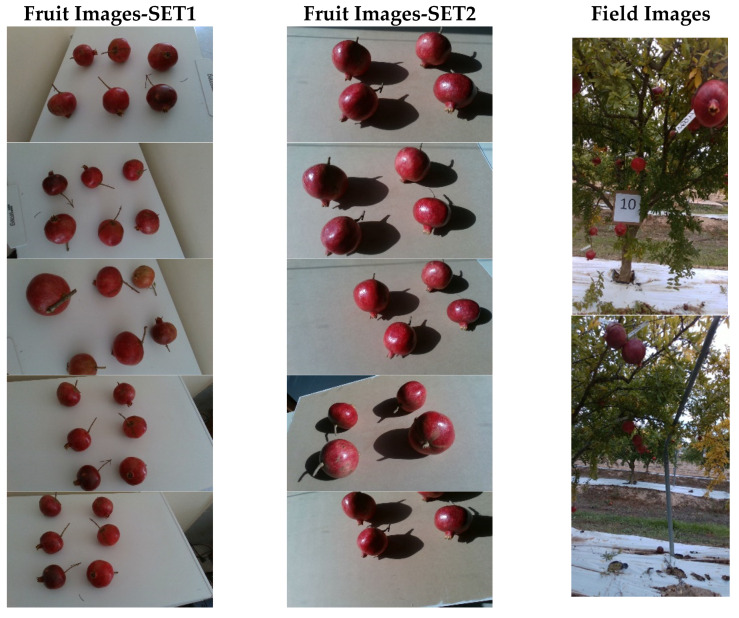
Sample images from the different data sets: controlled environment acquisitions under uniform (**left**) and non-uniform (**center**) exposure; field images acquired by the robotic platform (**right**).

Laboratory acquisitions of about 150 different pomegranates were performed with the same camera as the one onboard the robot, using the following two different setups: the first one, referred to as SET1, under uniform lighting conditions; the second one, referred to as SET2, under intense and non-uniform sun exposure. In both cases, fruits were arranged on a flat surface of neutral color, and spread well apart from each other. The reason for this split in the acquisitions is to insert an intermediate stage between the algorithm developed for indoor image labeling and the network that will be used for the segmentation of acquisitions in the field, as will be shown in the next section. Sample images from the different data sets are reported in Figure 2.

### 2.2. Image Segmentation

In the context of this study, image segmentation refers to separating fruits from non-fruit regions as a preliminary step before agricultural tasks such as fruit grading, counting or harvesting. To this end, a multi-stage transfer learning approach is developed, whereby a pre-trained network is initially tuned using fruit images that were acquired under controlled acquisitions, and then progressively extendedto more complex scenarios. The main advantage of the proposed approach is in automating the image annotation process required for network training, hence relieving the burden of having to use conventional manual labeling.

**Figure 3:**
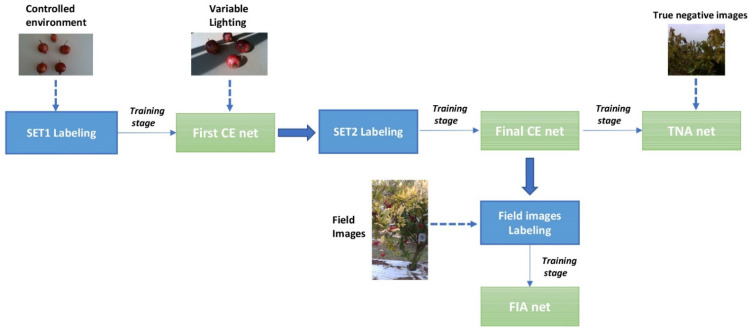
Pipeline of the image segmentation approach.

The overall image processing pipeline is shown in Figure 3. Firstly, fruit images acquired on a neutral background under controlled lighting conditions (SET1) are automatically labeled based on color thresholding, followed by morphological operations, and then fed to a pre-trained deep learning architecture (Stage 1). This process builds a new network that is referred to as *First Controlled Environment (CE) net*. The First CE net is, in turn, used to label additional fruit images that are still acquired from a neutral background but under natural lighting conditions (SET2) that could not be straightforwardly labeled based on color thresholds, mainly due to the presence of shadows. Labeled images from SET2 are then used, along with labeled images from SET1, to retrain the network (Stage 2) and deal with non-uniform lighting conditions, leading to the so-called *Final CE net*.

Finally, field images that were acquired by the robot are added to the training set for a final training stage (Stage 3). Two different kinds of field images are considered: images containing only non-fruit regions (i.e., leaves, branches and background), and images containing both fruit and non-fruit regions. In the first case, image masks are obtained directly by setting to 0 all the image pixels. For the labeling of field images, instead, the Final CE net is employed, followed by the use of morphological operations instead of classical manual annotation. This leads to two nets, referred respectively to as *True Negative Augmented (TNA)* net and *Field Image Augmented (FIA)* net. As will be shown in the experimental results section, the TNA helps to reduce false positives, whereas the FIA network leads to a reduction in false negatives.

#### 2.2.1. Network Architecture

**Figure 4:**
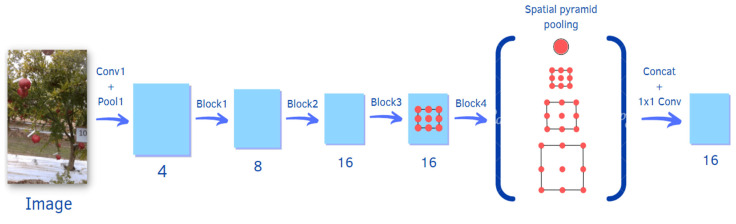
Block diagram of the DeepLabv3+ architecture using a set of 18 consecutive convolutional layers.

In this research, the DeepLabv3+ pre-trained deep neural network is used. In DeepLabv3+ [20], features are extracted from a backbone network, in this case a ResNet18; it processes the input image, along with a set of 18 consecutive convolutional layers, where a pre-trained version of the network trained on more than a million images from the ImageNet (http://www.image-net.org, accessed on 8 August 2022) database is adopted. The extracted features are input to an Atrous Spatial Pyramid Pooling (ASPP) network, which resamples the features at arbitrary resolutions in order to achieve the best pixel-by-pixel classification. The output of the ASPP network is passed through a 1 × 1 convolution to rearrange the data and obtain the final segmentation mask. A schematic representation of the network is depicted in Figure 4, which also shows its complexity at a glance. The strategy of transfer learning is thus applied to the proposed networks. Since the required number of the target classes is equal to 2, the last fully-connected layer of every deep network is downsized to output a binary classification. In this manner, the network is not initialized, and thus trained from the scratch.

#### 2.2.2. Controlled Environment (CE) Net

RGB images of harvested pomegranates located on a neutral background and acquired under uniform lighting conditions (SET1) are first fed to an algorithm using color-based thresholding, in order to obtain ground-truth masks. Labels at this step have several noise blobs in the background as well as some stems, which need to be removed. In order to improve the labels, morphological operations are applied to automatically clean all masked images. The following operations, based on the connected components, are applied to each binary mask:

1. Noise removal: connected components with areas that are below a threshold were eliminated.
2. Stem discarding: morphological erosion is used to separate the protruding stems of each pomegranate from the rest of the fruit. The resulting connected components are approximated by ellipses. Stems are thus removed by a thresholding operation on the ellipse’s eccentricity. The mask is finally restored by a dilation operation.
3. Hole-filling: a hole-filling operation is used to make labels uniform, even in the shaded areas.

**Figure 5:**
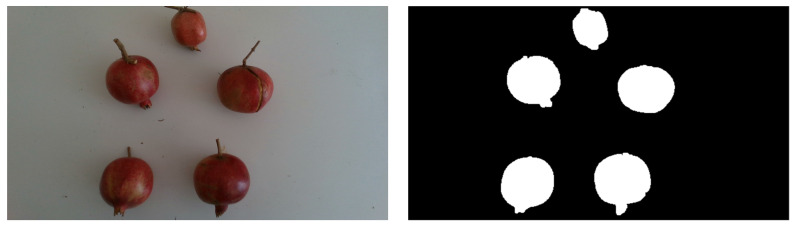
Sample image from SET1 (**left**) and the corresponding labeled image obtained by color threshold and morphological operations (**right**).

An example of a labeled image from SET1 is shown in Figure 5.

**Figure 6:**
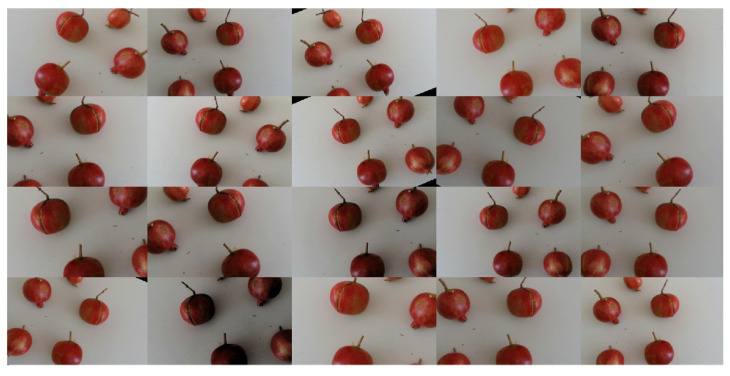
Data augmentation for the sample image of Figure 5. Twenty images are obtained by rotating, reflecting and varying contrast and exposure.

During the training phase, the more data that are available, the better. In order to satisfy this constraint, data augmentation is performed by rotating, reflecting and varying contrast and exposure in such ways that from each original image of SET1, 20 new images are obtained, as shown in Figure 6 for the sample case of Figure 5.

**Figure 7:**
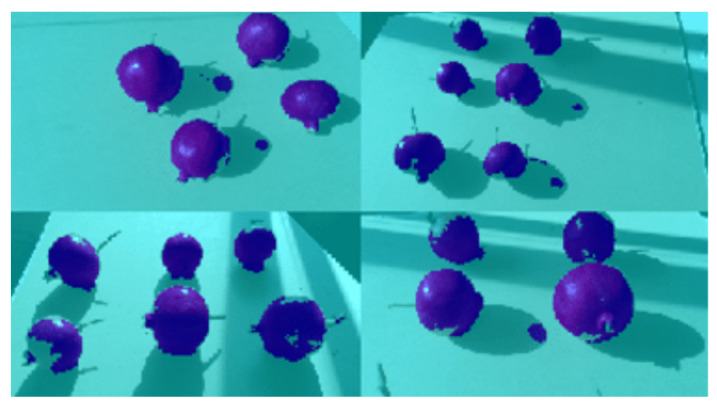
Four segmented images of SET2, in which each pixel recognized as a fruit by the First CE net is blue, while those in the background are cyan.

**Figure 8:**
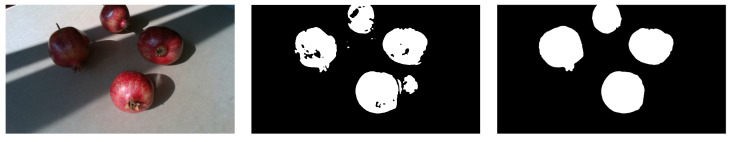
Results of labeling for a sample image of SET2: original image (**left**); labeled image obtained using the First CE net before (**center**) andafter morphological operations (**right**).

Labeled images are then used to tune a pre-trained network (Stage 1) and generate the *First Controlled Environment (CE) net*. The *First CE net* is then applied to produce image labels for images from SET2. Images from SET2 provide poor results when segmented using standard color-based filtering techniques, mainly as a result of the presence of shadows. Segmentation results for four sample images from SET2 are shown in Figure 7. This process facilitates the creation of labels that conventional color threshold tools are not able to obtain, due to the strong contrasts and chromatic distortions of the images. Also in this case, morphological operations are applied as previously described to improve the ground-truth masks that are used for subsequent training (see Figure 8 as an example).

**Figure 9:**
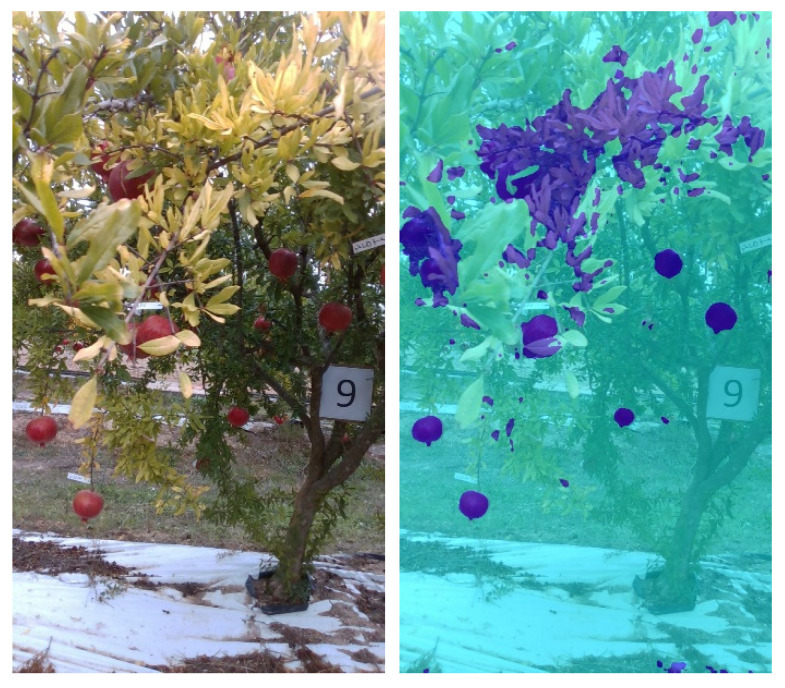
Final CE net result for a sample image acquired in the field. (**Left**): original image; (**right**): segmentation result.

Specifically, a new transfer learning stage (Stage 2) is performed using the full data set that was obtained from all the controlled environment acquisitions (both SET1 and SET2), resulting in the *Final CE net*. For simplicity’s sake, this net will be referred to as *CE net* in the following. As will be shown in Section 3, this network has an excessive sensitivity in RGB space when applied to field images, neglecting, in some cases, other parameters such as shape. This behavior is primarily evident in the presence of dry and yellowed leaves near the fruits, as is the case in Figure 9. The colors of those leaves tend to confuse the network, consequently increasing the number of false positives exponentially. For this reason, a further training stage is proposed (Stage 3), as described in the following.

#### 2.2.3. True Negative Augmented (TNA) Net

**Figure 10:**
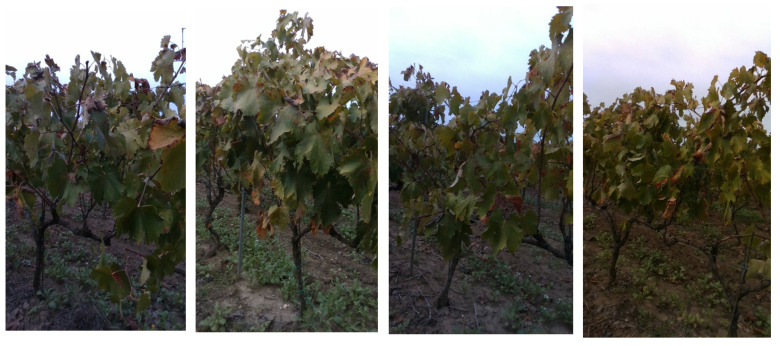
True negative images acquired in a vineyard used for TNA training.

**Figure 11:**
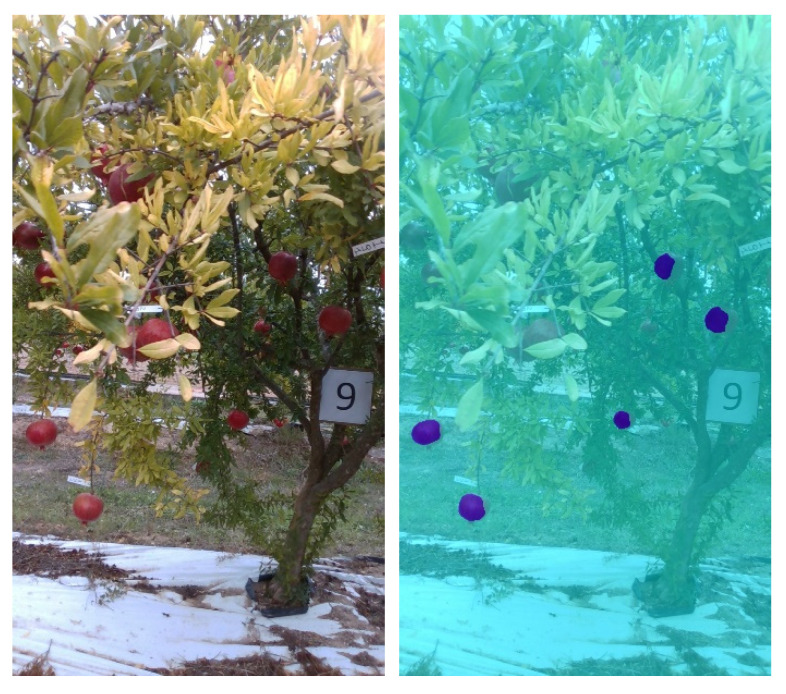
TNA result for the same image as Figure 9. (**Left)**: original image; (**right**): segmentation result.

In order to enhance the network capability of reducing false positives, true negative examples are added to labeled images from SET1 and SET2 as inputs for network training (Stage 3). True negative examples are images that contain only background information, such as fruitless images that were not necessarily acquired in the same crop type. In this case, images taken from a vineyard during the post-harvesting stage are used, as shown in the sample images in Figure 10. The resulting network is referred to as *True Negative Augmented (TNA)* net. In Section 3, it will be shown that by applying the TNA net to the segmentation of field images, false positives are drastically reduced, although at the cost of a reduction in true positives, as can be seen for the sample case shown in Figure 11.

#### 2.2.4. Field Image Augmented Network (FIA) Net

**Figure 12:**
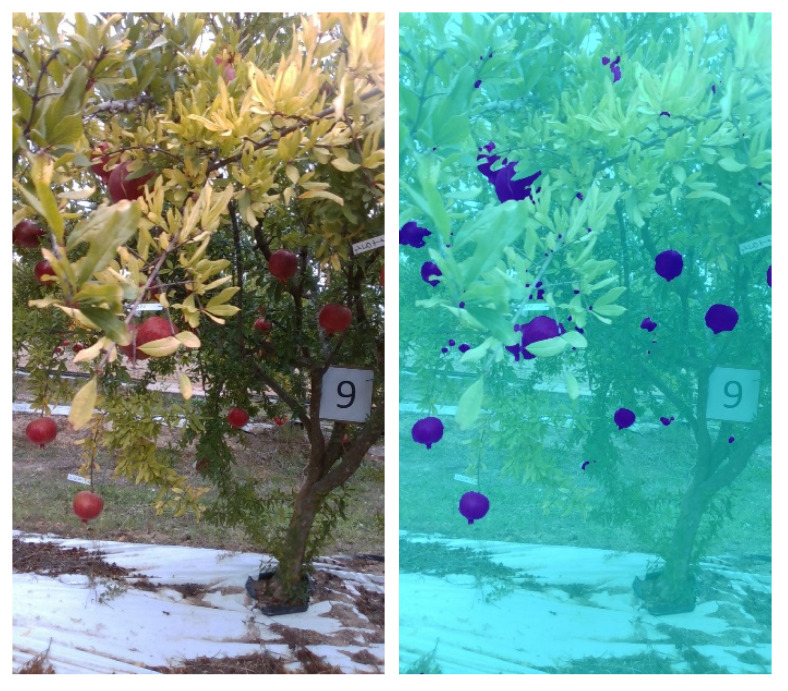
*FIA* result for the same image as Figure 9. (**Left**): original image; (**right**): segmentation result.

As an alternative strategy, the data set can be augmented using in-field image examples. Masks are generated from the frames of the first few seconds of field acquisition by applying the CE net. No morphological finishing operations are applied to the obtained labels. A new data set for training is then generated by adding these images to the labeled images from SET1 and SET2 for retraining the network (Stage 3). The resulting net, referred to as *Field Image Augmented Network (FIA)* net, leads to an improvement in system performance, reducing both false negatives and false positives, as can be seen for the case shown in Figure 12, and is discussed in detail in Section 3.

## 3. Results and Discussion

This section reports on the field validation of the proposed multi-stage transfer learning approach using the experimental setup described in Section 2. Quantitative metrics obtained from the CE, TNA and FIA networks are discussed. Results are also compared with a conventional approach using manually labeled field frames for training.

Each network is trained using different data sets that increase in complexity. The data sets used for training are summarized in Table 1. Specifically for the CE net, images from controlled environments enclosed in SET 1 and SET 2 are used after augmentation. The TNA network is trained by the addition of 100 true negative examples, including images of leaves and other background parts (e.g., sky regions and branches). It is worth noting that both the CE and the TNA net do not use images from the robot field acquisitions. A relatively small number of field images (i.e., 100), including both positive and negative examples of fruits acquired during the robot motion, are enclosed instead for the training of the FIA network. The CE, TNA and FIA networks are trained without requiring any manual labeling. Manually labeled field images are only used to compare the proposed approach with a conventional training procedure.

Once trained, the generalization ability of the networks is evaluated by applying the model to an independent (i.e., different from that used for training) data set that consists of 50 field images acquired during robot motion. Ground-truth labels for these images are obtained via manual labeling.

### 3.1. Evaluation Metrics

Pixel-wise accuracy is measured by comparing ground truth and predicted information. Specifically, having defined as *TP* the number of true positives (i.e., pixels correctly classified as fruit), *TN* as the number of true negatives (i.e., pixels correctly classified as non-fruit), *FN* as the number of false negatives (i.e., pixels incorrectly classified as non-fruit) and *FP* as the number of false positives (i.e., pixels incorrectly classified as fruit), precision (*P*), recall (*R*) and the F1-score are recovered as follows:

(1) $$P=\frac{TP}{TP+FP}$$

(2) $$R=\frac{TP}{FN+TP}$$

(3) $$F1-\mathrm{score}=2\cdot \;\frac{P\cdot R}{P+R}$$

In addition, the following metrics are computed:

- *Global Accuracy*, the ratio of correctly classified pixels, regardless of class, to the total number of pixels. This metric allows for a quick and computationally inexpensive estimate of the percentage of correctly classified pixels.
- *Intersection over union* (*IoU*), also known as the Jaccard similarity coefficient, is the most commonly used metric. It provides a measure of statistical accuracy that penalizes false positives. For each class, *IoU* is the ratio of correctly classified pixels to the total number of true and predicted pixels in that class, as shown in the following:

(4) $$IoU=\frac{TP}{TP+FP+FN}$$

For each image, mean *IoU* is the average *IoU* score of all classes in that particular image. For the aggregate data set, mean *IoU* is the average *IoU* score of all classes in all images.

- *Weighted IoU*, is the average *IoU* of each class weighted by the number of pixels in that class. This metric is used when images have disproportionally sized classes, in order to reduce the impact of errors in the small classes on the aggregate quality score.

### 3.2. Segmentation Performance

The performance of the CE, TNA and FIA networks is reported in Table 2, in terms of precision, recall and F1-scores. These metrics are computed for each image and then averaged over the entire test set.

These results show the performance of the segmentation algorithm using different training sets. Referring to Table 2, the CE net shows reasonably good recall values amounting to 79.06%, with a relatively low precision of 48.10%. These results were due to a high number of false positives that mainly related to the misclassification of dry yellow leaves, as shown in the sample case in Figure 8. Conversely, the TNA network shows an increment in the precision that reached a value of 96.87%, thanks to the addition of true negative examples to the training set, at the expense of recall, which dropped from 79.06% to 43.25%. Thus, the network no longer confuses fruit with other elements in a scene; however, at the same time, it loses information by missing several fruits. Both CE and TNA nets show comparable F1-score values of 55.79% and 56.97%, respectively. A significant improvement was achieved with the FIA net, in which the training set was enriched with a few images acquired in the field and directly labeled through the CE network, following the pseudo-labeling learning strategy. The FIA net guarantees the best performance, with precision and recall values of 93.33% and 81.49%, respectively, resulting in an F1-score of 86.42%.

**Figure 13:**
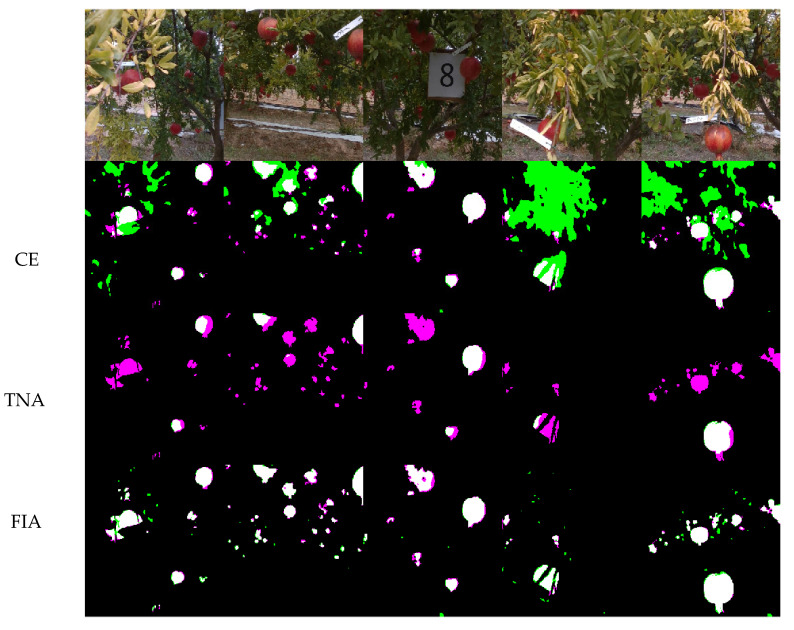
Comparison among segmentation results obtained from the three different approaches. White pixels represent true positives, black pixels represent true negatives, pink pixels represent false negatives and green pixels represent false positives.

Figure 13 shows the segmentation results obtained from the different networks for some sample field images, with white pixels representing true positives, black pixels representing true negatives, magenta pixels representing false negatives and green pixels representing false positives. The progressive reduction in misclassification passing from the CE to FIA net can be clearly seen.

**Figure 14:**
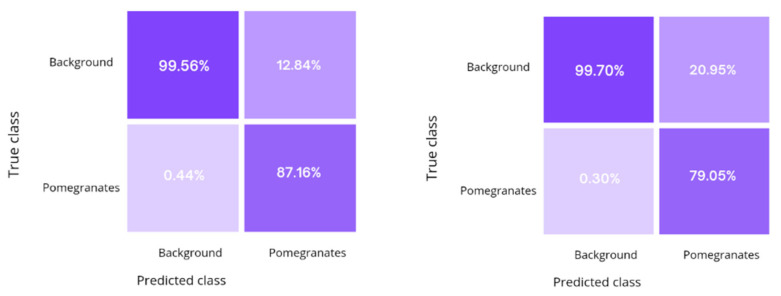
Confusion matrices for the manual labeling network (**left**) and the FIA network (**right**).

The proposed multi-stage approach was compared with a conventional manual labeling procedure. The results are collected in Table 3. The confusion matrices that summarize the percentage of correct and incorrect predictions over the entire test set are also shown in Figure 14. The proposed approach leads to comparable results to standard training with manually labeled examples, with fairly consistent results in terms of accuracy and IoU, and with a relatively low decrement of about 4.0% in the F1-score, but with the advantage of relieving the manual labeling burden.

The computational efficiency of the FIA network was also evaluated by segmenting the entire field image data set, using an Nvidia RTX 2080 Ti GPU and an Intel (R) Core (TM) i7-4790 CPU @ 3.60 GHz, showing that the processing time attests to about 0.15 s per frame (less than the acquisition frame rate of 6 Hz) This would make it feasible the adoption of the proposed approach for real-time processing onboard farm robots.

## 4. Conclusions

In this paper, a multi-stage transfer learning approach has been developed to segment field images of pomegranate fruits acquired by an agricultural robot, without the need for a time-consuming manual labeling process. Each learning stage leads to a different network, ranging from a network that was trained using images acquired under controlled conditions (CE network), up to a network (FIA network) that incorporates field images as well. Results obtained from experimental tests have been presented, and show that despite the low quality of the input images, the proposed methods can segment field images achieving an F1-score of 86.42% and IoU of 97.94%, using the DeepLabv3+ architecture. The obtained performance is comparable with those of a standard learning approach, without the burden of having to use manual annotation.

### Future Work

The methods discussed in the paper use a consumer-grade camera (worth a few hundred Euros) as the only sensory input. This choice proved to be a good trade-off between performance and cost-effectiveness. An obvious improvement would be to use higher-end depth cameras available on the market. Future efforts will be devoted to further improving the proposed framework, e.g., using other pre-trained nets, and to extend the scope of the system to yield prediction or automatic size estimation of fruits. In this respect, the use of 3D data will be specifically investigated in order to improve classification results, and to recover further information on fruit morphology. In addition, the proposed framework could be enhanced for fruit control by the farmer robot. Finally, the portability of the system to different crops (e.g., vineyards), or for different fruit maturation stages, will be evaluated.

## Figures and Tables

**Table 1:** Data sets used for training.

	SET 1 Images	SET 2 Images	True Negative Images	Field Acquisition Images	Training Images after Augmentation
**CE**	174	136	-	-	6200
**TNA**	174	136	100	-	8200
**FIA**	174	136	-	100	8200
**Manual labeling**	-	-	-	410	8200

**Table 2:** Precision, recall and F1-scores for multi-stage transfer learning using DeepLabv3+ architecture.

Networks	Precision	Recall	F1-Score
**CE**	48.10%	79.06%	55.79%
**TNA**	96.87%	43.25%	56.97%
**FIA**	93.33%	81.49%	86.42%

**Table 3:** Comparison between FIA net and the manual labeling net (precision, recall, F1-score, accuracy and IoU).

	Precision	Recall	F1-Score	Global Accuracy	Mean Accuracy	Mean IoU	Weighted IoU
**FIA Network**	93.33%	81.49%	86.42%	98.93%	89.38%	86.16%	97.94%
**Manual Labeling**	91.36%	88.88%	89.91%	99.09%	93.36%	88.68%	98.29%

## Data Availability

The data presented in this study can be made available on request.
